# The Relative Location of the Major Femoral Nerve Motor Branches in the Thigh

**DOI:** 10.7759/cureus.3882

**Published:** 2019-01-14

**Authors:** Branten J Page, Oliver D Mrowczynski, Russell A Payne, Sarah E Tilden, Hector Lopez, Elias Rizk, Kimberly Harbaugh

**Affiliations:** 1 Anatomy, Penn State Hershey Medical Center, Hershey, USA; 2 Neurosurgery, Penn State Hershey Medical Center, Hershey, USA

**Keywords:** peripheral nerve surgery, surgical anatomy, femoral nerve

## Abstract

In peripheral nerve surgery, repair of the femoral nerve (FN) requires identification of normal nerve elements both proximal and distal to the level of the injury. We identified FN branches to the sartorius (SRT) and quadriceps muscles in 16 embalmed specimens and calculated the length of each branch to its point of entry into its respective muscle. The SRT and rectus femoris (RF) muscles were mobilized but not transected to mimic the surgical approach. Ratios of the length of each motor branch as a unit of the total length of the thigh, defined as the FN at the inguinal ligament to the superior margin of the patella were also calculated. The proximal branch to RF spanned a ratio of .19 ± .11 (mean ± standard deviation) from the FN at the inguinal ligament to its endpoint. The ratio of the distal branch to the RF was .29 ± .08. The ratio of the proximal SRT branch was .20 ± .05. The distal branch to SRT was located at a ratio of .43 ± .11. The proximal branch to vastus lateralis (VL) was .26 ± .08. The distal branch to VL was .39 ± .07. The ratio of the motor branch to vastus intermedius (VI) was .30 ± .05. Lastly, the branch to vastus medialis (VM) was .55 ± .06. The motor branch to SRT frequently emerged as a bifurcation of itself and saphenous nerve within the adductor canal. Knowledge of the relative location of the motor branches of the FN in the thigh can be helpful to surgeons during the nerve exploration and repair.

## Introduction

In peripheral nerve surgery, repair of injured nerves requires identification of the normal nerve elements both proximal and distal to the level of injury. In the case of the femoral nerve (FN), identification of the branching pattern just distal to the inguinal ligament and the distal motor branches where many FN injuries requiring surgical repair occur can be extremely difficult. Injuries in this location often have associated vascular injuries with prior repair and subsequent significant scarring, complicating surgery. 

We sought to identify the FN branches in the thigh at their point of muscle entry and calculate the location relative to the length of the thigh.

## Materials and methods

Anatomy and morphology

We identified and dissected the FN unilaterally (nine left and seven right) on 16 embalmed cadavers, (10 females and six males) from the Penn State College of Medicine with ages at death ranging from 71 to 95 years (mean = 84).

The FN was located at the inguinal ligament (FNIL). The sartorius (SRT) and RF muscles were mobilized but not divided and vascular structures were preserved in order to more closely approximate the surgical approach. Upon identification of the FNIL, its motor branches to the SRT, RF, vastus medialis (VM), vastus intermedius (VI) and vastus lateralis (VL) were traced and dissected to the point of entrance into the muscle. The saphenous nerve was also identified and isolated from structures within the adductor canal. We did not attempt to identify the pectineus motor branch given its location posterior to the femoral sheath and its minor clinical significance in FN repair.

Measurements

Measurements of the distance between the bony anterior superior iliac spine (ASIS) and the ipsilateral pubic tubercle (PT) and between the ASIS to the FNIL were taken. The ratio of the relative location of the FNIL was calculated. The distances from the FNIL to the superior border of the patella and between the FNIL to the motor point of the SRT and each of the quadriceps muscles were also measured. A ratio of the FNIL to motor point distance relative to the FNIL to patella distance was then calculated for each muscle. A pin was placed in the FNIL and a string passed to the point at which each motor branch reached the anterior compartment muscles. The measurement for distance down the leg as a percentage was (FNIL-Motor point)/(FNIL-Patella). The measurements were determined by a combination of calipers and rulers.

## Results

The mean medial-lateral distance from the ASIS to the PT was 13.03 ± 1.40 cm (mean ± standard deviation) (Table 1). Measurements can be found in Table 1.

**Table 1:** Measurements between anatomical landmarks ASIS = anterior superior iliac spine; PT = pubic tubercle; FN = femoral nerve; FNIL = femoral nerve at the inguinal ligament; prox = proximal; dist = distal; SRT = sartorius; RF = rectus femoris; VL = vastus lateralis; VI = vastus intermedius; VM = vastus medialis; st. dev. = standard deviation Note: all measurements are in centimeters

Cadaver #	Sex	Leg	ASIS-PT	ASIS-FN	FN-Patella	FN-prox.SRT	FN-dist.SRT	FN-prox.RF	FN-dist.RF	FN-prox.VL	FN-dist.VL	FN-VI	FN-VM
1	F	R	15.5	5.5	41	9	18	7	11	12.5	18.5	10.5	26.5
2	M	L	13.5	6	45	11	21.5	11	15.5	14	18.5	11	25
3	M	R	15	7.5	40	6	27	5.5	10	10	19.5	16	26
4	F	R	14	7	41	8	11	9	13	9.5	13.5	13	21
5	F	L	11.5	7	38	8	21	7	11	10.5	17	10.5	17.5
6	M	R	14	8	40	4.5	13	7.5	11.5	9	12	15.5	23
7	F	L	14	7.5	37	7.5	12	5.5	10	8	13.5	9.5	19
8	M	R	14	7	44	9	21	8.5	13.5	10	17	not identified	28
9	M	R	12	7	39	4.5	12	9	12	8	12	10	20
10	F	L	11.5	7	38	10.5	18.5	7	11.5	11	19	13	21
11	F	L	11	4	39	8	15	8.5	12	11	16	11.5	21.5
12	F	L	12	6	39	6	16	6	10	10	15.5	9.5	21
13	F	R	14	5	42	12	22	9	12.5	11	12.5	13.5	24.5
14	F	L	13	6	39.5	8	17	8.5	13	11.5	16	13	22
15	M	L	11.5	6	40	8.5	18.5	7	9	11	15	10.5	20.5
16	F	L	12	6.5	40.5	7	12.5	8	10.5	11.5	17.5	11.5	18.5
mean			13.03125	6.4375	40.1875	7.96875	17.25	7.75	11.625	10.53125	15.8125	11.9	22.1875
st dev.			1.4	1.03	2.11	2.12	4.54	1.47	1.64	1.54	2.52	2.04	3.04

The average length from the FNIL to the patella was 40.19 ± 2.11 cm. Table 2 shows ratios of FNIL to the motor point when visualized from lateral to medial.

**Table 2:** Ratios of FNIL to motor point (Superior-Inferior axis) SRT = Sartorius; RF = Rectus femoris; VL = Vastus lateralis; VM = Vastus medialis; VI = Vastus intermedius; prox = proximal; FNIL = femoral nerve at the inguinal ligament * Given as a percentage of the FNIL – Patellar distance

Nerve Branch	Motor Point Distance from FNIL*	Motor Point Location on Muscle
SRT-prox	20%	Medial surface
SRT-distal	43%	
RF-prox	19%	Deep surface after wrapping around medial aspect of muscle.
RF-distal	29%	
VL-prox	26%	Anteromedial surface
VL-distal	39%	
VM	55%	
VI	30%	Anterior surface

**Figure 1:**
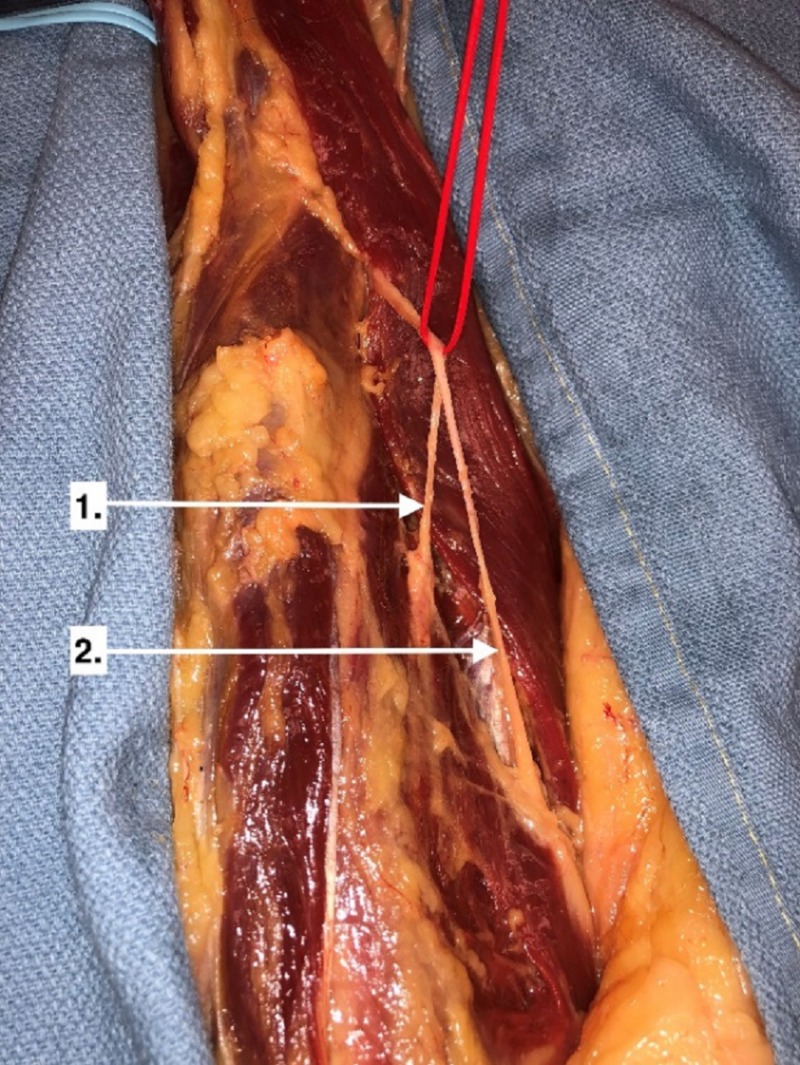
Motor branch to vastus medialis (1), saphenous nerve (2) This image illustrates a dissection of the right anterior thigh demonstrating the common branching of vastus medialis motor branch from the saphenous nerve.

The RF and SRT muscles both received two branches. The motor point for the proximal branch of both was similarly located at approximately 8 ± 2.12 cm from the FNIL and approximately 20% of the distance from the FNIL to the superior patella. The distal branches for the SRT and RF were located 17.25 ± 5.21 cm and 11.63 ± 1.64 cm from the FNIL, corresponding to 43% and 29% of the FNIL to patella distance, respectively.The VM received one branch at 22.19 ± 3.04 cm from the FNIL corresponding to 55% of the FNIL to patella distance. The nerve ran along the anteromedial surface of the muscle and often arose as a branch from the saphenous nerve (Figure 1).

The VL and VI motor branches passed to the muscles posterolaterally under the dense fascial septum of the RF muscle with the descending branches of the lateral circumflex femoral vessels. The VL had multiple branches at the muscle entrance. We measured the distance to the proximal and distal-most branches, 10.53 ± 1.54 cm and 15.81 ± 2.52 cm, corresponding to a ratio of approximately 0.26 and 0.39 of the FNIL-patella distance, respectively. VI received one primary branch 11.90 ± 2.04 cm from FNIL corresponding to a ratio of 0.30 of the FNIL to patella distance. For cadaver 13, the nerve branch to the VI could not be detected presumably because it was severed during the dissection. In most cases, further branching could be visualized within a centimeter of the terminal insertion of the nerve branches to the various muscles. The measurements and ratios are listed in Tables 1 and 2, respectively.

The anatomical side and surface at which each muscle received nerve branches remained consistent among cadavers. The RF received its main nerve branches on its deep surface, with the nerve wrapping under the muscle from the medial side. The SRT received its proximal and distal branches on its medial surface. The VL and VM received their branches on the anteromedial surface and VI received branches on the anterior surface. On cadaver three, the nerve branch to the VI branched from one of the VL branches prior to reaching the VL muscle entrance point.

## Discussion

**Figure 2:**
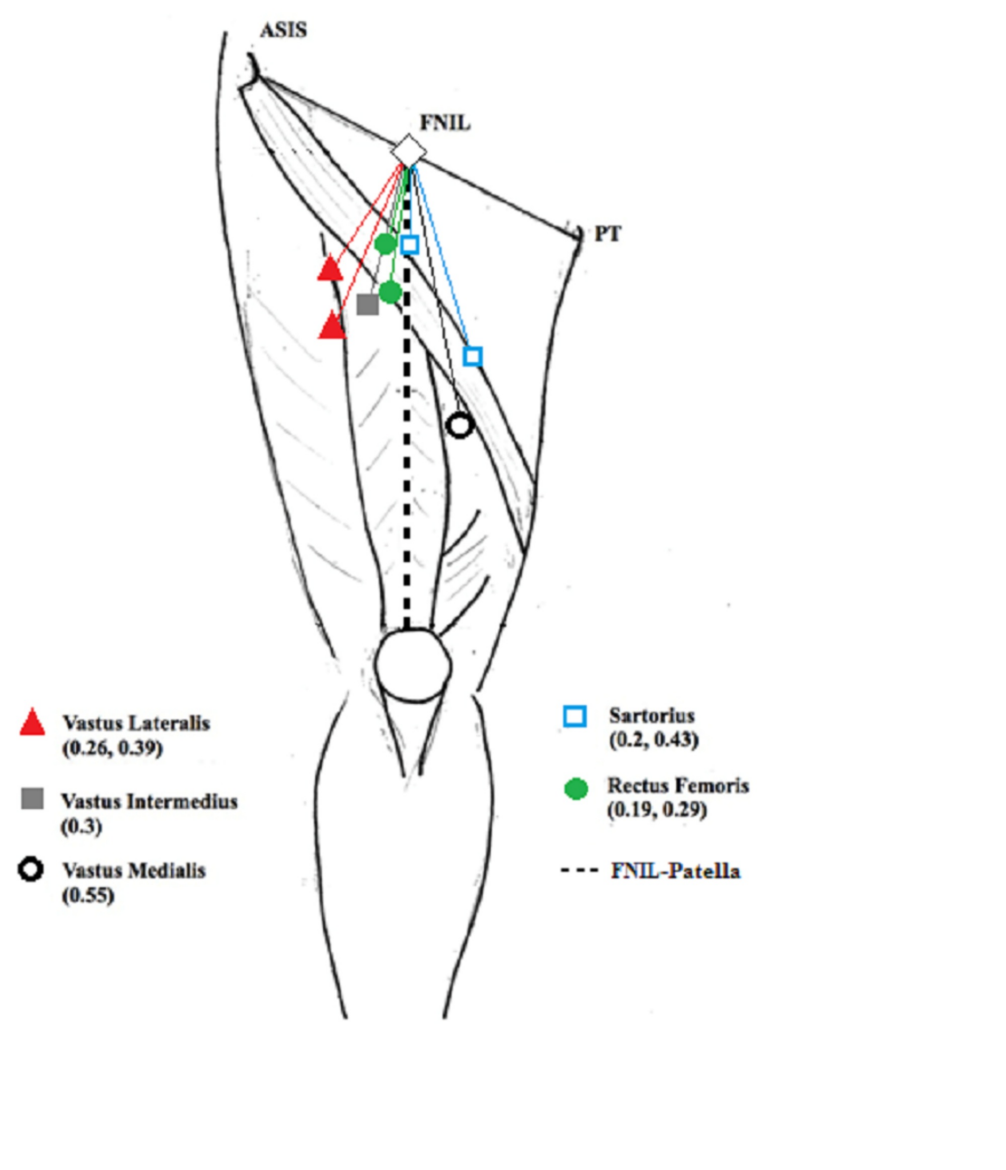
Diagram of the right thigh indicating the relative locations of the femoral motor branches Numbers in parentheses indicate the ratio. ASIS = anterior superior iliac spine; FNIL = femoral nerve at the inguinal ligament; PT = pubic tubercle

For surgical repair of a nerve, identification of normal proximal and distal segments of the injured nerve allows for evaluation of nerve regeneration and repair in those cases where the nerve is severed or has a high-grade non-regenerating lesion. Given the branching of the FNIL, identification of normal distal nerve can be challenging especially in those cases involving an associated vascular injury and/or prior vascular repair. For this reason, knowledge of the relative location of the FN motor branches to their respective muscles of innervation can be useful to surgeons attempting to repair the FN after injury (Figure 2).

While prior anatomical descriptions are available, the SRT and RF muscles are removed in order to identify the motor branches to the vastus musculature [1]. This would not be a viable option in the surgical setting. We found that with the careful mobilization of the SRT and RF muscles, the vastus branches can be identified at their muscle entrance sites while preserving the RF and SRT branches. 

As noted in ours and other studies, the FN is situated centrally along the inguinal ligament between the pubic tubercle (PT) and the ASIS [2]. This information is helpful in those cases where the femoral artery has been bypassed and the pulse may not be in its normal location just medial to the FN.

The FNSRT and RF muscles were found to have two branches each. This is similar to the findings of others, although some report a single motor branch to the RF that branches into two separate sub-branches [3-5]. The motor branches of SRT and RF enter the medial side of the muscles and for both, the proximal branches were approximately one-fifth of the distance between the FNIL and the patella or thigh length. This corresponds well to findings of Sung et al. who noted the rectus branch 19% distally along the inguinal ligament to patella line [3]. The distal branches were located at approximately 30% and 40% down the length of the thigh for the RF and SRT, respectively. The number and location of these branches should be considered during the mobilization of the muscles in order to prevent their injury during surgical repair as well as facilitate nerve grafting. 

In our series and others, the branch to VM was the most distally located approximately half way down the thigh [6]. The branch was well defined running along the antero-medial surface of the muscle with the saphenous nerve and could be mobilized well proximally. Our nerve branch corresponds to the major medial branch noted by Thirangama [7]. We did not reliably see a branch corresponding to their smaller lateral branch. 

The VL branches were also easily identified lateral and deep to the RF muscle coursing laterally with the descending branches of the lateral circumflex femoral vessels lying at a distance between 25% and 40% distally down the thigh. Our findings correspond to other authors [8-9]. The VI branch was somewhat more challenging to locate and in one case we were unable to identify the VI motor branch. Additionally, we noted that the VI branch arose from a VL branch in one cadaver. Branches to VI from VL and VM have been reported previously [8, 10]. The VI motor branch entered the muscle at a point distally 30% of the distance from FNIL to the patella. This closely matches the 0.29 ratio for VI motor point location reported by Albert et al. [10].

The identification of the nerve to VI was in part more difficult given its deep and more medial location compared with the VL branches as we were trying not to sever the RF branches on the medial side of the muscle and thus approached the VI branches from the lateral side of the RF. Similarly, the distal to proximal dissection of the VL branches and the branch to the VI was challenging in those specimens with thicker RF fascia or septum between the RF and VL. In the face of prior injury, this would likely add further complexity and should be taken into consideration in the clinical setting.

Anatomical texts often describe the separation of the FN into an anterior or medial division that innervates the SRT muscle and gives off the medial and intermediate cutaneous nerves of the thigh, and a posterior or lateral division that gives off motor branches to the quadriceps muscles and the saphenous nerve [1]. A clear demarcation of the FN into these divisions would be helpful from a reconstructive standpoint and we initially sought to assess this. Unfortunately, the FNIL in our study was typically flattened and without a reliable demarcation of the nerve into distinct divisions. This is often seen clinically and as has been noted previously [2]. In addition, the branching was not consistent. For this reason, we did not attempt to systematically detail the relative branch location of the FNs at the inguinal ligament. In general, the proximal SRT branch and cutaneous thigh branches did come off more medially and the VL and VI branches were more centrally or laterally located. The RF and VM branches were more variably located. However, these findings were not consistent enough to suggest preferential grafting of the lateral portion of the nerve at the inguinal ligament. In fact, Gustafson et al. found that the fascicles to the quadriceps muscles that are conducive to standing were more centrally and dorsally located compared to the SRT, RF, and cutaneous branches [2].

Our study has some limitations. We did not have multiple independent investigators to measure the distances which would have strengthened the validity of the results. Future clinical and cadaver studies attempting to identify the location of the motor branches prior to exploration to assess the validity of our findings are needed and planned. Overall, we feel that the ratio measures are easier for clinicians to remember and for this reason are more useful than numerical values that may vary with patient size. 

## Conclusions

The FN motor branches in the thigh could be identified with mobilization but preservation of the SRT and RF muscles. Our findings were similar to prior reports with more aggressive muscle dissection. The medial location of the RF and SRT branches should be considered when mobilizing these muscles in the surgical setting. Knowledge of the relative location of the motor branches of the FN in the thigh can be helpful to surgeons during nerve exploration and repair. 
